# CrusTF: a comprehensive resource of transcriptomes for evolutionary and functional studies of crustacean transcription factors

**DOI:** 10.1186/s12864-017-4305-2

**Published:** 2017-11-25

**Authors:** Jing Qin, Yaohua Hu, Ka Yan Ma, Xiaosen Jiang, Ching Hei Ho, Ling Ming Tsang, Lefei Yi, Ricky Wai Tak Leung, Ka Hou Chu

**Affiliations:** 1 Simon F. S. Li Marine Science Laboratory, School of Life Sciences, The Chinese University of Hong Kong, Shatin, New Territories Hong Kong, China; 2 Shenzhen Research Institute, The Chinese University of Hong Kong, Shenzhen, 518057 China; 3 College of Mathematics and Statistics, Shenzhen University, Shenzhen, 518060 China; 4 Department of Information Engineering, The Chinese University of Hong Kong, Shatin, New Territories Hong Kong, China; 5 Institute of Marine Biology, National Taiwan Ocean University, Keelung, Taiwan; 6 College of Marine Life and Fisheries, Huaihai Institute of Technology, Lianyungang, 222005 China

**Keywords:** Transcription factor, Crustacea, Transcriptome, Transcriptional regulatory function, Evolution

## Abstract

**Background:**

Crustacea, the second largest subphylum of Arthropoda, includes species of major ecological and economic importance, such as crabs, lobsters, crayfishes, shrimps, and barnacles. With the rapid development of crustacean aquaculture and biodiversity loss, understanding the gene regulatory mechanisms of growth, reproduction, and development of crustaceans is crucial to both aquaculture development and biodiversity conservation of this group of organisms. In these biological processes, transcription factors (TFs) play a vital role in regulating gene expression. However, crustacean transcription factors are still largely unknown, because the lack of complete genome sequences of most crustacean species hampers the studies on their transcriptional regulation on a system-wide scale. Thus, the current TF databases derived from genome sequences contain TF information for only a few crustacean species and are insufficient to elucidate the transcriptional diversity of such a large animal group.

**Results:**

Our database CrusTF (http://qinlab.sls.cuhk.edu.hk/CrusTF) provides comprehensive information for evolutionary and functional studies on the crustacean transcriptional regulatory system. CrusTF fills the knowledge gap of transcriptional regulation in crustaceans by exploring publicly available and newly sequenced transcriptomes of 170 crustacean species and identifying 131,941 TFs within 63 TF families. CrusTF features three categories of information: sequence, function, and evolution of crustacean TFs. The database enables searching, browsing and downloading of crustacean TF sequences. CrusTF infers DNA binding motifs of crustacean TFs, thus facilitating the users to predict potential downstream TF targets. The database also presents evolutionary analyses of crustacean TFs, which improve our understanding of the evolution of transcriptional regulatory systems in crustaceans.

**Conclusions:**

Given the importance of TF information in evolutionary and functional studies on transcriptional regulatory systems of crustaceans, this database will constitute a key resource for the research community of crustacean biology and evolutionary biology. Moreover, CrusTF serves as a model for the construction of TF database derived from transcriptome data. A similar approach could be applied to other groups of organisms, for which transcriptomes are more readily available than genomes.

**Electronic supplementary material:**

The online version of this article (10.1186/s12864-017-4305-2) contains supplementary material, which is available to authorized users.

## Background

Transcription factors (TFs), regulators of gene transcription, are critical for most if not all biological processes. They possess DNA-binding domains (DBDs) that can recognize specific DNA sequences to mediate the TF-DNA interaction. By binding to functional DNA elements, such as promoter, enhancer, silencer, and insulator, TFs can activate or suppress the transcription of their target genes and precisely control the cell phenotypes and functions. The TF repertoires of many species throughout the tree of life have been available in several TF databases, most of which identify TFs from genome sequences. For example, DBD (transcription factor prediction database) predicts TFs from 930 completely sequenced genomes, in Archaea, Bacteria, and Eukaryota [1]. CIS-BP (Catalog of Inferred Sequence Binding Preferences) covering 290 eukaryotic genomes accrue DNA binding motifs based on the experimental identification and computational inference [2]. Other TF databases that focus on specific taxonomic groups are also mainly based on genome sequences. For instance, AnimalTFDB recorded TFs from 65 animals, mostly vertebrates [3], while FlyFactorSurvey [4] and FactorBook [5] focus on the binding specificity of TFs of fruit fly, human, and mouse. However, as only a few crustacean genomes have been sequenced to date, among the mentioned TF databases, only CIS-BP covers two crustacean species, while DBD contains one. Consequently, critical knowledge concerning crustacean TFs, including their sequences, functions and evolution, have been rarely explored.

Crustaceans are a diverse group of animals of ecological and commercial importance all over the world; including many species of high economic values in fisheries and aquaculture, particularly the decapods such as crabs (Brachyura), lobsters (Astacidea and Achelata), crayfishes (Astacidea) and shrimps (Caridea and Dendrobranchiata). The total production of crustacean farming in 2015 has increased to nearly 14 million tons, with an average annual increase rate of 3.75% in recent years [6]. About half of all crustacean products are produced by aquaculture, and the other half relies on capture fishery [6]. The proportion of crustaceans among all aquaculture animals has increased from less than 5% before 2000 to close to 10% in 2015, valued at over US$38 billion [7]. The escalating harvest of wild stocks of commercially important decapod crustaceans for fishery and aquaculture has also incited public concern about the sustainability and the detrimental environmental impact of such practice. A few studies have already documented the negative effects of crustacean fishery and aquaculture on the environment and crustacean biodiversity [8, 9]. Besides the economic species, many small crustacean species are important components in various ecosystems. Many of them, for instance, the krill (Euphausiacea) and copepods (Copepoda), are a major food resource for many marine faunae, serving as important trophic links between the primary producers and the macrofauna [10]. Others, such as amphipods (Amphipoda) and *Daphnia* (Cladocera), are often used as bioindicators to assess the impact of human activity and environmental changes on ecosystems and biodiversity [11–13]. Research on the growth, reproduction, and development of crustaceans is particularly crucial to fisheries, aquaculture, biodiversity conservation and environmental protection. Given the importance of TFs in these biological processes, investigations on crustacean TFs become urgent for the improvement of crustacean fisheries and aquaculture, and mitigation of biodiversity degradation and loss.

Our current knowledge on crustacean TFs is mainly derived from low-throughput experiments, and restricted to only a few TFs in a few species [14–17]. A system-wide exploration on the repertoire of crustacean TFs will shed light on the complexity and diversity of crustacean transcriptional regulatory systems. Fortunately, despite the lack of genome sequence for most crustacean species, a large repertoire of transcriptomes of many crustacean species emerged in recent years provides us the opportunity to predict TFs from assembled transcripts on a transcriptome-wide scale. We collected the publicly available and newly sequenced crustacean transcriptomes in our laboratory and searched all TFs in the transcriptome assemblies. To disseminate our results, we constructed a database CrusTF, in which coding sequences (CDSs), protein sequences, DBDs, DNA binding motifs and phylogeny of crustacean TFs could be freely accessed by researchers. Compared to current TF databases containing only a few crustaceans, CrusTF includes TFs from 170 crustacean species. It is the first TF database derived from transcriptome data. It will serve as a model to fill the knowledge gap of TF genes throughout the tree of life for those species of which transcriptomes are more readily available than genomes.

## Construction and content

CrusTF is a database of crustacean TFs mainly derived from de novo transcriptome assemblies. It has explored a comprehensive collection of crustacean transcriptomes to identify transcribed crustacean TFs. It allows users to search TFs with keywords matching TF names or TF identifiers, to select species and TF family of interest, or to search by Blast tools with their own TF sequences. Free batch download is available for the TF CDSs, protein sequences and domain sequences of selected species or TF families. Besides TF sequences, CrusTF also contains functional and evolutionary information of crustacean TFs.

### Database implementation

CrusTF implements a Linux-Apache-MySQL-PHP (LAMP) system. All data were saved in MySQL database, including TF sequences, TF information, domains, species information, and motifs. The web is constructed based on CodeIgniter, a powerful PHP framework. CodeIgniter provides an Application Programming Interface (API) to connect the web to MySQL database. We also used JavaScript libraries including jQuery (2.2.0), jQuery-labelauty and some additional plugins to perform dynamic web services.

### Data resources

Crustacean transcriptomes were downloaded from two public databases, National Center for Biotechnology Information (NCBI) Transcriptome Shotgun Assembly (TSA) database (https://www.ncbi.nlm.nih.gov/genbank/tsa/) and Short Read Archive (SRA) database (https://www.ncbi.nlm.nih.gov/sra). The detailed information of all transcriptomes from SRA and TSA is summarized in Additional file 1: Tables S2 and S3, respectively. As of January 2017, our transcriptome collection composes of 919 and 122 crustacean transcriptome samples curated from SRA and TSA databases, respectively (Additional file 1: Tables S2 and S3). 37 RNA-seq samples of 31 crustacean species generated in our laboratory were also included in the current version of CrusTF (Additional file 1: Table S1 and unpublished data of Ma et al.). Additional file 1: Table S1 lists the transcriptome data sources of each crustacean species available in CrusTF. Besides, crustacean genes, including those derived from low-throughput experiments, were downloaded from GenBank (https://www.ncbi.nlm.nih.gov/genbank/) (Additional file 1: Table S4).

### Data processing

For transcriptomes in TSA, assembled contigs were directly downloaded from the database. For transcriptome data in SRA, raw reads of RNA sequencing (RNA-seq) were downloaded. To standardize the data processing procedure, only RNA-seq data generated using Illumina sequencers were selected. Quality of raw reads was assessed by FastQC (https://www.bioinformatics.babraham.ac.uk/projects/fastqc/). Adapters were trimmed by Trimmomatic [18], allowing two seed mismatches to tolerate sequencing errors in adapter sequences. Subsequences of reads with low quality (average quality score lower than 20) were removed. Trimmed reads shorter than 50 nucleotides were also deleted. Processed raw reads were assembled de novo using Trinity version 2.4.0 with the default setting to obtain contigs of transcript sequences [19]. Potentially contaminated sequences from bacteria, virus or archaea were filtered by Kraken [20]. Contigs from multiple transcriptomes of the same species are clustered and further assembled by TGICL with an identity cutoff of 0.94 to reduce redundancy [21]. CDSs and amino acid sequences were deduced from assembled contigs, as well as sequences from GenBank, with Transdecoder [22].

### Functional annotation of TFs

DNA binding domains (DBDs) were identified by scanning all crustacean proteins with HMMs of known DBDs from Pfam database [23] by PfamScan that implements HMMER3 [24]. Predicted TFs with DBDs were compared to known TFs well annotated in CIS-BP and named by the best matched known TFs.

DNA binding motif of each TF was inferred as described previously [2]. In brief, DBDs of crustacean TFs were compared with those of TFs with known binding motifs, and similarity between crustacean TFs and known TFs was calculated. Based on the observation on co-evolution of DBD sequences and their DNA binding motifs, it has been reported that the TF binding motifs of a TF could be inferred from those of homologous TF DBDs when the identity between the two DBDs is greater than a threshold [2]. The thresholds of all TF families could be found in CIS-BP (file cisbp_1.02.tf_families.sql in the package from “Download MySQL Tables” in http://cisbp.ccbr.utoronto.ca/bulk.php). Known TF binding motifs of metazoan TFs were downloaded from CIS-BP [2], JASPAR [25], UniPROBE [26] and hPDI [27]. DNA binding motif of crustacean TFs could be inferred when their DBDs were highly similar to TFs with DNA binding motifs that have been detected experimentally.

The confidence level of a predicted TF was estimated using several criteria: 1) the percentage of the top hit that matches the predicted TF, E-value, Blast score and sequence identity when Blast to the protein database SwissProt, 2) the bit-score and E-value of PfamScan, 3) the number of homologs found in other crustacean species, 4) the number of transcriptome samples from which the TF was detected. Crustacean TFs of each family were ranked according to the Blast E-value, PfamScan E-value, the number of crustacean homologs and the number of supported samples, respectively. The TFs were ranked according to the four criteria. The ranks of each TF imply the confidence of the predicted TF.

### Evolutionary analysis

The DBD sequences of the crustacean TFs and TFs of 117 other animals in each TF family were aligned with Clustal Omega, which is a fast and scalable tool for multiple amino acid sequence alignment [28]. Approximately-maximum-likelihood phylogenetic tree of each TF family was reconstructed with FastTree with JTT + CAT model [29]. Trees were visualized by R package ggtree [30] and iTOL (Interactive Tree Of Life) [31]. Users can manipulate the trees interactively via iTOL.

## Utility and discussion

**Fig. 1:**
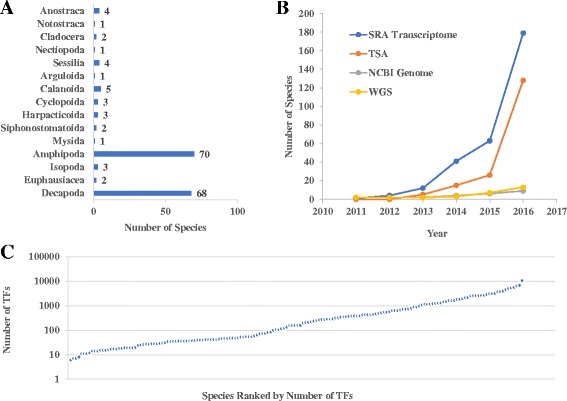
Statistics of CrusTF. **a** Number of species belonging to 15 orders of Crustacea. **b** Increase in the number of crustacean species of which transcriptomes or genomes have been published. All four databases belong to National Center for Biotechnology Information (NCBI). SRA Transcriptome: Transcriptomes (RNA-seq) in Short Read Archive; TSA: Transcriptome Shotgun Assembly database; NCBI Genome: NCBI genome database; WGS: Whole Genome Shotgun database. **c** Number of TFs identified in each species

The crustacean TFs in CrusTF are classified according to the species and functional domain types. Users can choose a species of interest from the species list to browse all crustacean TFs identified from its transcriptomes. In the current version, CrusTF has a total of 170 crustacean species (Table 1), of which only two species (*Daphnia pulex* and *Artemia franciscana*) are included in other TF databases (DBD and CIS-BP) based on genome sequences. Our collection covers 15 crustacean orders (Fig. 1a). Two major orders are Decapoda and Amphipoda, which have 68 and 70 species in our collection, respectively. The former is the order containing many economic crustacean species, like crabs, lobsters, crayfishes, and shrimps (http://www.fao.org/fishery/collection/asfis/en), while the latter contains many species for environmental monitoring [32]. Even though other orders only have 1 to 5 species, they are usually the most representative species in those orders. The high coverage of species in this subphylum allows the direct comparison of TF sequences among different crustaceans. So CrusTF provides the function of searching similar TFs in other species for each TF. Homolog search against crustacean TFs from transcriptomes, GenBank, and TFs from genomes of other animals could be easily achieved by click on the “Search” buttons in the main page of each TF. Users can investigate how homologous TFs are changed among different species.

**Table 1:** Crustacean species available in CrusTF

Species	#Transcriptome
Class Branchiopoda	
Subclass Sarsostraca	
Order Anostraca	
*Artemia franciscana*	10
*Artemia salina*	2
*Artemia sinica*	2
*Artemia tibetiana*	2
Subclass Phyllopoda	
Order Notostraca	
*Triops newberryi*	1
Order Cladocera	
*Daphnia magna*	55
*Daphnia pulex*	21
Class Remipedia	
Order Nectiopoda	
*Xibalbanus tulumensis*	1
Class Maxillopoda	
Subclass Thecostraca	
Order Sessilia	
*Amphibalanus amphitrite*	3
*Megabalanus volcano*	4
*Tetraclita japonica*	5
*Tetraclita squamosa*	4
Subclass Branchiura	
Order Arguloida	
*Argulus siamensis*	1
Subclass Copepoda	
Order Calanoida	
*Calanus finmarchicus*	14
*Calanus glacialis*	1
*Calanus sinicus*	1
*Eurytemora affinis*	9
*Pseudocalanus acuspes*	14
Order Cyclopoida	
*Eucyclops serrulatus*	2
*Lernaea cyprinacea*	2
*Paracyclopina nana*	2
Order Harpacticoida	
*Tigriopus californicus*	28
*Tigriopus japonicus*	1
*Tigriopus* sp. 1 SL-2012	1
Order Siphonostomatoida	
*Caligus rogercresseyi*	10
*Lepeophtheirus salmonis*	1
Class Malacostraca	
Subclass Eumalacostraca	
Order Mysida	
*Neomysis awatschensis*	1
Order Amphipoda	
*Acanthogammarus godlewskii*	1
*Asprogammarus rhodophthalmus*	1
*Baikalogammarus pullus*	1
*Boeckaxelia carpenterii*	1
*Boeckaxelia potanini*	1
*Brachyuropus grewingkii*	1
*Brandtia latissima*	1
*Carinurus bicarinatus*	1
*Cornugammarus maximus*	1
*Crypturopus inflatus*	1
*Dorogostaiskia parasitica*	1
*Echinogammarus veneris*	2
*Echiuropus macronychus*	1
*Eucarinogammarus wagii*	1
*Eulimnogammarus cruentus*	2
*Eulimnogammarus cyaneus*	1
*Eulimnogammarus czerskii*	1
*Eulimnogammarus marituji*	1
*Eulimnogammarus messerschmidtii*	1
*Eulimnogammarus similis*	1
*Eulimnogammarus* sp. gam16.4	1
*Eulimnogammarus* sp. gam2quest	1
*Eulimnogammarus testaceus*	1
*Eulimnogammarus ussolzewii*	1
*Eulimnogammarus verrucosus*	1
*Eulimnogammarus violaceus*	1
*Eulimnogammarus viridulus*	1
*Eulimnogammarus vittatus*	1
*Gammarus chevreuxi*	1
*Gammarus fossarum*	1
*Gammarus lacustris*	1
*Garjajewia dershawini*	1
*Gmelinoides fasciatus*	1
*Gondogeneia antarctica*	1
*Heterogammarus sophianosii*	1
*Homalogammarus brandtii*	1
*Hyalella azteca*	3
*Hyalellopsis carinata*	1
*Hyalellopsis costata*	1
*Hyalellopsis grisea*	1
*Hyalellopsis setosa*	1
*Hyalellopsis stebbingi*	1
*Linevichella vortex*	1
*Macrohectopus branickii*	1
*Macropereiopus parvus*	1
*Macropereiopus wagneri*	1
*Melita plumulosa*	1
*Micruropus glaber*	1
*Micruropus parvulus*	1
*Micruropus wahlii*	1
*Odontogammarus calcaratus*	1
*Ommatogammarus albinus*	1
*Ommatogammarus flavus*	1
*Oxyacanthus curtus*	1
*Oxyacanthus flavus*	1
*Oxyacanthus sowinskii*	1
*Pachyschesis branchialis*	2
*Palicarinus puzyllii*	1
*Pallasea cancelloides*	2
*Pallasea cancellus*	1
*Pallasea grubei*	1
*Pallasea* sp. gam7.3	1
*Pallaseopsis kessleri*	1
*Pandorites podoceroides*	1
*Parapallasea borowskii*	1
*Parapallasea wosnessenskii*	1
*Pentagonurus dawydowi*	1
*Poekilogammarus pictoides*	1
*Sluginella kietlinskii*	1
*Talitrus saltator*	2
Order Isopoda	
*Armadillidium nasatum*	2
*Armadillidium vulgare*	10
*Asellus aquaticus*	1
Order Euphausiacea	
*Euphausia crystallorophias*	1
*Meganyctiphanes norvegica*	1
Order Decapoda	
Suborder Dendrobranchiata	
*Penaeus aztecus*	1
*Penaeus merguiensis*	1
*Penaeus monodon*	10
*Penaeus vannamei*	41
Suborder Pleocyemata	
Infraorder Caridea	
*Antecaridina lauensis*	1
*Caridina rubella*	1
*Halocaridinides trigonophthalma*	1
*Macrobrachium nipponense*	7
*Macrobrachium rosenbergii*	29
*Metabetaeus lohena*	1
*Metabetaeus minutus*	1
*Neocaridina denticulata*	1
*Palaemon argentinus*	1
*Palaemon carinicauda*	1
*Pandalus latirostris*	1
Infraorder Astacidea	
*Astacus astacus*	2
*Astacus leptodactylus*	5
*Cherax cainii*	1
*Cherax destructor*	1
*Cherax quadricarinatus*	7
*Homarus americanus*	2
*Nephrops norvegicus*	1
*Pacifastacus leniusculus*	1
*Procambarus clarkii*	7
Infraorder Achelata	
*Sagmariasus verreauxi*	1
Infraorder Anomura	
*Calcinus laevimanus*	1
*Coenobita clypeatus*	1
*Coenobita* sp.	1
*Pagurus bernhardus*	1
*Petrolisthes lamarckii*	1
Infraorder Brachyura	
*Anatolikos japonicus*	1
*Calappa philargius*	1
*Callinectes sapidus*	4
*Callinectes similis*	1
*Cancer borealis*	1
*Carcinus aestuarii*	2
*Carcinus maenas*	13
*Carinoplax longimana*	1
*Erimacrus isenbeckii*	1
*Eriocheir sinensis*	28
*Eriphia smithii*	1
*Gecarcoidea lalandii*	1
*Geothelphusa eucrinodonta*	1
*Grapsus albolineatus*	1
*Hyas araneus*	6
*Leptodius* sp.	1
*Leucosiidae* sp.	1
*Liocarcinus depurator*	2
*Lydia annulipes*	1
*Macrophthalmus abbreviatus*	1
*Majidae* sp.	1
*Matuta victor*	1
*Mictyris brevidactylus*	1
*Necora puber*	8
*Ocypode ceratophthalmus*	1
*Ovalipes punctatus*	1
*Ozius rugulosus*	1
*Parasesarma pictum*	1
*Pinnotheridae* sp.	1
*Plagusia squamosa*	1
*Portunus pelagicus*	1
*Portunus trituberculatus*	5
*Ranina ranina*	1
*Scopimera bitympana*	1
*Scylla olivacea*	4
*Scylla paramamosain*	4
*Tymolus uncifer*	1
*Xenograpsus testudinatus*	1

When compared to the number of available crustacean genomes, the number of crustacean species with transcriptome data grows much faster in recent years (Fig. 1b). Thus, identification of TFs from de novo assembled transcriptomes would be a much more efficient approach for species without available genomes and an important supplement to the current methods based on whole genome sequences. However, as shown in Table 1, since the data volumes for different species vary, the coverage of transcriptomes may be quite different among species. The number of total unique contigs varies from 1102 to 641,047, due to the variations of library preparation methods, sequencing throughput and the number of samples. Therefore, the number of TFs predicted from different species ranges from 6 to 10,535 (Fig. 1c). This is because of the limitations of the transcriptomic approach. First, transcriptomes are usually incomplete when the sequencing throughput is low or the RNA samples used cannot cover a wide range of different tissues and conditions. When a gene is not expressed in the sampled conditions, it would not be sequenced. Thus, these species have less predicted TFs. Secondly, de novo assembly of transcriptome may generate many false transcripts, which could be filtered out by comparing to known genes or protein features. Yet we did not filter them out, because filtering assembled transcripts based on current knowledge may lead to loss of novel TFs that may be very important. Thus, we keep all predicted TFs and provide the information of evidence that support them (See Construction and content), from which users can easily estimate the reliability of the predicted TF and also have the opportunity to explore new TFs for further investigation. Despite its limitations, our transcriptomic approach is still an efficient and valuable way to predict TFs, especially when the genomes of many species are not available.

**Fig. 2:**
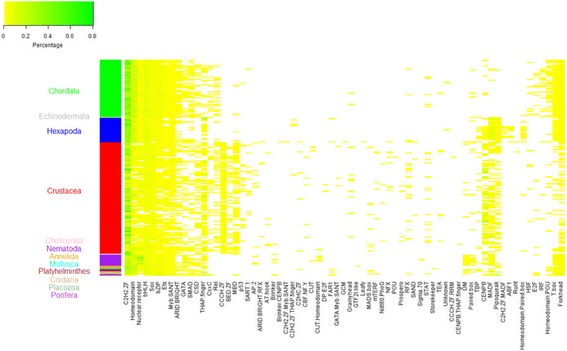
TF families in crustaceans compared to those in other animals. Colors in the figure show the percentage of TFs in each TF family over all predicted TFs in a species (white, <1%; yellow to green, 1–100%). Each row is a species and each column is a TF family. Side bar highlights the taxa. Many TF families on the left that are prevalently detected in metazoans were detected in most crustaceans. Several TFs families, such as families with Zinc finger CCCH domain (CCCH ZF) and BED zinc finger (BED ZF), show extensive expansion in crustaceans. Some TF families with distinct DBD combinations may represent putative TFs unique to this animal group and have not been characterized in other TF databases

In the current version, CrusTF contains 131,941 and 8502 TFs of crustacean species from transcriptomes and GenBank, respectively. They are classified into 63 TF families according to the DBDs or DBD combinations detected in their sequences (Fig. 2). Detailed information of TF families are listed in and Additional file 1: Table S5. Many TF families that are prevalently detected in metazoans were detected in most crustaceans. Interestingly, crustaceans have distinct patterns of TF family composition when compared to other animals (Fig. 2). Several TFs families, such as families with Zinc finger CCCH domain and BED zinc finger, show extensive expansion in crustaceans. Some TF families with distinct DBD combinations may represent putative TFs unique to this animal group and have not been characterized in other TF databases (Fig. 2). Users can browse the TFs of a certain TF family by selecting it from the TF family list. And they can also download all TF sequences of a family from a species of interest or all crustacean species in the “Download” page.

To facilitate the users to understand the phylogenetic relationships among crustacean DBDs and DBDs from other animals, the phylogenetic tree of each DBD type is available in the “Trees” page. These trees visualize the phylogenetic relationships among DBDs from our 170 crustaceans and 117 other animals of 9 phyla from Porifera to Chordata. Based on the phylogenetic relationships of DBDs, CrusTF has inferred the DNA binding motif for each TF from their closest TFs with motifs derived from experimental studies. Users can browse the motif information on the web page of each TF and download the motifs for further prediction of downstream targets.

## Conclusion

In summary, CrusTF is the first TF database derived from transcriptome data. It uncovers the specific pattern of the transcriptional regulatory system of crustaceans and the diversity of TFs in this important group of animals. This database will constitute a key resource for the research community of crustacean biology and evolutionary biology. Given the importance of TF information in functional studies on transcriptional regulatory systems of crustaceans, it will facilitate the research works on growth, reproduction, and development of crustaceans, and subsequently benefit studies on crustacean fisheries, aquaculture, biodiversity conservation and environmental protection.

## Additional file

Additional file 1: Table S1. List of crustacean transcriptome data. **Table S2.** Crustacean transcriptome data collection from Short Read Archive. **Table S3.** Crustacean transcriptome data collection from Transcriptome Shotgun Assembly. **Table S4.** Crustacean TFs from GenBank. **Table S5.** List of the 63 TF families identified in the crustacean species. (XLSX 336 kb)

## Abbreviations

API: Application programming interface
CDS: Coding sequence
CIS-BP: Catalog of inferred sequence binding preferences
DBD: DNA-binding domain
iTOL: Interactive tree of life
LAMP: Linux-Apache-MySQL-PHP system
NCBI: National Center for Biotechnology Information
RNA-seq: RNA sequencing
SRA: Short read archive
TF: Transcription factor
TSA: Transcriptome shotgun assembly database
ZF: Zinc finger
